# Comparison of surveillance-based metrics for the assessment and monitoring of disease detection: simulation study about type 2 diabetes

**DOI:** 10.1186/s12874-017-0328-2

**Published:** 2017-04-11

**Authors:** Ralph Brinks, Annika Hoyer, Deborah B. Rolka, Oliver Kuss, Edward W. Gregg

**Affiliations:** 1 German Diabetes Center, Leibniz Institute for Diabetes Research at the Heinrich-Heine-University Düsseldorf, Institute for Biometry and Epidemiology, Auf’m Hennekamp 65, Düsseldorf, 40225 Germany; 2 University Hospital at the Heinrich-Heine-University Düesseldorf, Hiller Research Unit for Rheumatology, Moorenstrasse 5, Düsseldorf, 40225 Germany; 3 Centers for Disease Control and Prevention, Division of Diabetes Translation, Atlanta, Georgia, USA; 4 German Center for Diabetes Research (DZD), Munich–Neuherberg, Germany

**Keywords:** Compartment model, Incidence, Prevalence, Diabetes, Chronic disease, Undiagnosed disease, Case-finding, Screening

## Abstract

**Background:**

Screening and detection of cases are a common public health priority for treatable chronic conditions with long subclinical periods. However, the validity of commonly-used metrics from surveillance systems for rates of detection (or case-finding) have not been evaluated.

**Methods:**

Using data from a Danish diabetes register and a recently developed illness-death model of chronic diseases with subclinical conditions, we simulate two scenarios of different performance of case-finding. We report different epidemiological indices to assess case-finding in both scenarios and compare the validity of the results.

**Results:**

The commonly used ratio of detected cases over total cases may lead to misleading conclusions. Instead, the ratio of undetected cases over persons without a diagnosis is a more valid index to distinguish the quality of case-finding. However, incidence-based measures are preferable to prevalence based indicators.

**Conclusion:**

Prevalence-based indices for assessing case-finding should be interpreted with caution. If possible, incidence-based indices should be preferred.

**Electronic supplementary material:**

The online version of this article (doi:10.1186/s12874-017-0328-2) contains supplementary material, which is available to authorized users.

## Background

Chronic conditions like coronary heart disease, type 2 diabetes, hypertension, cancer, osteoporosis, and dementia frequently have long periods wherein the condition is undiagnosed. Although the specific policies related to active population screening are sometimes controversial, the prevalence of these undiagnosed conditions can be substantial and the period of undiagnosis is sometimes a missed opportunity to implement preventive care to reduce the risk of subsequent morbidity. Because of the lack of direct data on case-finding, population surveys with information on prevalence of diagnosed and undiagnosed cases are often incorporated into indirect indices to make inferences about levels of case-finding that are occurring. For example, national monitoring of public health efforts in the U.S. includes the tracking of proportion of cases of total diabetes [1], chronic kidney disease [2], and hypertension [3], who are aware of their condition or have been diagnosed. Similar metrics are used in diverse international settings to assess the degree of awareness, treatment and diagnosis of hypertension [4], hyperlipidemia [5], and diabetes [6]. Although these metrics are intended to assess the degree of case detection that occurs in clinical and public health settings, the validity of these surrogates of the rate of case-finding has not been evaluated. In this analysis we examine common approaches to using cross-sectional data and find that the choice of metric can yield vastly different conclusions about trends in detection, with some yielding misleading conclusions.

In this article, we use a recently developed multi-state model and simulate two different scenarios about diabetes case-finding. Then, we apply and compare different metrics to assess the case-finding in the two scenarios.

## Methods

Based on data from the national Danish diabetes register on diagnosed diabetes, we simulate two different scenarios about case-finding, in one scenario the prevalence of undetected disease *increases* over time, and in another scenario the prevalence of undetected disease *decreases* over time. For brevity, we will denote the two scenarios as PU^+^ and PU^−^. Here, PU means *prevalence of undiagnosed* disease, and the + and – signs indicate the upward and downward trend in time, respectively.

For the simulation we use a previously published multi-state model. We then apply different surveillance-based indices to the PU^−^ and PU^+^ scenarios and compare the results of different indices.

After a brief description of the multi-state model, we describe the different indices for assessing case-finding. Then, details of the simulation are presented.

### Multi-state model

**Fig. 1:**
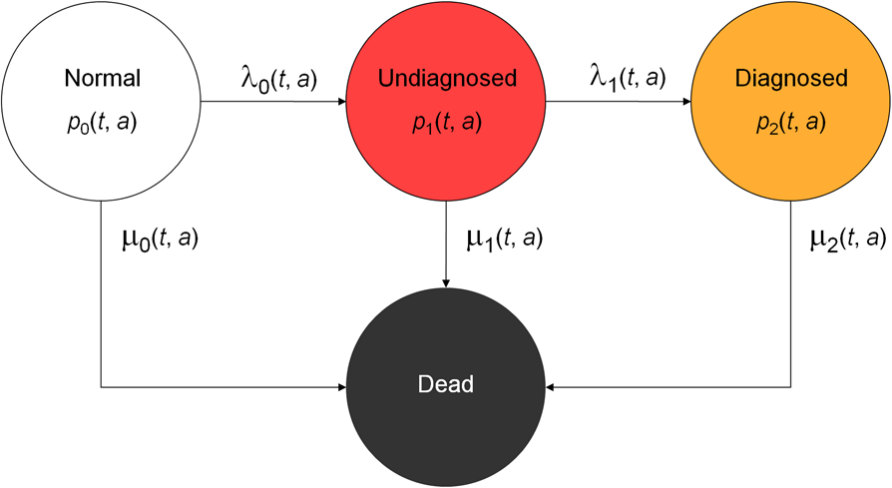
Chronic disease model with four states according to [7]. Persons in the state *Normal* are healthy with respect to the disease under consideration. After onset of the disease, they change to state *Undiagnosed* and later to the state *Diagnosed*. The absorbing state *Dead* can be reached from all other states. The numbers of persons in the states and the transition rates depend on calendar time *t* and age *a*

Recently, we described an illness-death model with a state of being *Undiagnosed* preceding the *Diagnosed* state (Fig. 1) [7]. At birth, each person is in the *Normal* state (here we just consider diseases acquired after birth). During the life course, the person may contract the disease and enter the *Undiagnosed* state. In a screening program, or as the disease becomes symptomatic, or during some routine medical examinations, the disease may be diagnosed and the person transitions to the *Diagnosed* state. Persons may die during any of these three states. Since we are considering chronic conditions only, we assume that backward steps from the *Undiagnosed* state to the *Normal* state are not possible. Similarly, there are no backward steps from the *Diagnosed* to the *Undiagnosed* state.

The transition rates *λ*_*ℓ*_(*t*,*a*), *ℓ*=0,1, and *μ*_*k*_(*t*,*a*), *k*=0,1,2, in the model and the percentages of persons in the states, the prevalences, are related by a two-dimensional system of partial differential equations [7]. As in [7] let *p*_*k*_(*t*,*a*) denote the fraction of persons aged *a* at time *t* in state *k*. In epidemiological settings time *t* is also called period. For example, *p*_2_(*t*,*a*) is the fraction of persons in the population who are aged *a* at time *t* and are in the *Diagnosed* state (*k*=2). It can be calculated as $p_{2}(t, a) = \tfrac {N_{2}(t, a)}{N(t, a)}$ where *N*_*k*_(*t*,*a*) denotes the number of people in state *k*, *k*=0,1,2, aged *a* at time *t* and *N*(*t*,*a*)=*N*_0_(*t*,*a*)+*N*_1_(*t*,*a*)+*N*_2_(*t*,*a*). Similary, the prevalence of the undiagnosed disease (*p*_1_) is defined as $p_{1}(t, a) = \tfrac {N_{1}(t, a)}{N(t, a)}.$

The system of partial differential equations governing the compartment model shown in Fig. 1 is:

1 $$ \begin{aligned} {}\left(\frac{\partial}{\partial t} \,+\, \frac{\partial}{\partial a}\right) p_{1} \,=\, \lambda_{0} \, (1 \,-\, p_{2}) \,-\, (\lambda_{0}\,+\,\lambda_{1} \,+\, \mu_{1} - \mu_{0}) \, p_{1} + p_{1} \, z  \end{aligned}  $$

2 $$ \begin{aligned} {}\left(\frac{\partial}{\partial t} + \frac{\partial}{\partial a}\right) p_{2} = - (\mu_{2} - \mu_{0}) \, p_{2} + \lambda_{1} \, p_{1} + p_{2} \, z, \end{aligned}  $$

where *z*=*p*_1_ (*μ*_1_−*μ*_0_)+*p*_2_ (*μ*_2_−*μ*_0_).

The rate *λ*_0_ can be interpreted as the *true incidence* or *total incidence* of type 2 diabetes. It describes how many persons develop diabetes in the considered population – irrespective of whether diabetes is diagnosed or not. The total incidence *λ*_0_ is affected by the aetiologic risk profile of the population. If risk factors become more prevalent in the considered population over time, the rate *λ*_0_ will increase accordingly. Vice versa, if risk factors become less frequent, *λ*_0_ will go down.

The rate *λ*_1_ describes the transition from the undiagnosed state to the diagnosed state. While *λ*_0_ is affected by aetiological factors, *λ*_1_ mainly depends on societal factors, e.g., medical progress in detecting the disease, awareness of patients and physicians, reimbursement of diagnostic testing etc. Epidemiologists are interested in both rates, *λ*_0_ as it reflects the risk profile of a population and *λ*_1_ as a societal construct. However, if an epidemiologist refers to “the incidence” of a chronic disease and the undiagnosed state is ignored, this “incidence” essentially refers to *λ*_1_. Hence, it allows little inference about the changes of risk profiles in the population.

### Indices for assessing case-finding

For better clarity, we categorize the indices on whether they are based on prevalence or transition rates in the multi-state model. The last category refers to an index, which is combined from prevalence and transition rates.

#### Indices based on the prevalence

The simplest index is the prevalence of the undiagnosed disease (*p*_1_). A high or an increasing value of *p*_1_ is intuitively considered to be unfavourable. For example, if *p*_1_ is 5% in a specific age group at a specific point in time, and is 10% two years later, then there is an (unfavourable) accumulation of undetected cases during these two years.

The second index to approach case-finding, is the proportion of detected cases from the total cases [1], i.e.,

3 $$ \omega_{1} = \tfrac{p_{2}}{p_{1}+p_{2}}.  $$

The reciprocal of *ω*_1_ describes the factor the diagnosed cases have to be multiplied with to obtain the number of all cases of the chronic disease. If *ω*_1_ equals 0.5, for instance, this means that for each detected case there is one undetected case. Obviously, it holds 0≤*ω*_1_≤1. A high value in *ω*_1_ is usually interpreted as advantageous with respect to case-finding [1].

Similarly, it may be useful to consider the proportion

4 $$ \omega_{2} = \tfrac{p_{1}}{p_{0}+p_{1}}.  $$

This proportion relates the number of persons in the undiagnosed state to all persons who do not have a diagnosis, i.e., the healthy and the undiagnosed. The idea behind the measure *ω*_2_ is that case-finding can be thought of the task of distinguishing persons from a pool consisting of healthy and undiagnosed persons. This pool of healthy and undiagnosed persons may be seen as the *search space*. The search space is subject to the activities of case-finding. Once an undiagnosed person is identified as a case, this person gets a diagnosis and is removed from the search space henceforth. As the disease under consideration is chronic, there is no way back into the search space, i.e., no remission can occur. In contrast to *ω*_1_, the figure *ω*_2_ just refers to the persons who are at risk for a possible diagnosis. Thus, the fraction of persons with a diagnosis does not play a role for *ω*_2_. The reciprocal of *ω*_2_ is the average number of persons without a diagnosis a physician must see to meet one undiagnosed case.

Again, it holds 0≤*ω*_2_≤1. Ideally, *ω*_2_ is 0, i.e., all undetected cases become diagnosed and are removed from the search space. The closer *ω*_2_ approaches 1, the more the search space is dominated by the undiagnosed persons. Thus, a lower value of *ω*_2_ is advantageous in assessing case-finding. This is consistent with the interpretation of *ω*_2_ being the reciprocal of the average number of persons without diagnosis a physician must meet to have one undiagnosed case. The lower *ω*_2_ is, the greater the reciprocal is. Hence, a lower *ω*_2_ implies a higher average number a physician must meet to find an undiagnosed case. This means, case-finding worked well in the time before the physician met these persons.

#### Indices based on the transition rates

Apart from the indices based on the prevalences, we may consider figures based on the transitions in the model. In [7] we used the detection rate ratio $\text {DR} = \tfrac {\lambda _{1}}{\lambda _{0}},$ which relates the instantaneous risk (hazard) of transitioning to the diagnosed state to the risk of becoming an undetected case. As it is unlikely to be diagnosed immediately after entering the undiagnosed state, we introduce a delay parameter *γ*, *γ*≥0, and define

$$\text{DR}_{\gamma}(t, a) = \frac{\lambda_{1}(t + \gamma, a + \gamma)}{\lambda_{0}(t, a)}. $$

It holds DR=DR_0_, and in [7] we have shown that DR determines if the prevalence of the undiagnosed disease is lowering or rising. The condition

5 $$ \text{DR}(t, a) < \frac{p_{0}(t, a)}{p_{1}(t, a)} + \frac{\mu(t, a)-\mu_{1}(t, a)}{\lambda_{0}(t, a)}  $$

is equivalent with increasing *p*_1_ in (*t*,*a*), i.e., *p*_1_(*t*,*a*)<*p*_1_(*t*+*δ*,*a*+*δ*) for small *δ*>0.

Another important index is the fraction of healthy persons aged *a* at time *t* who become incident undiagnosed cases at time *t* and die within *γ*>0 time units without ever obtaining a diagnosis. As these persons do not have a diagnosis, they never were treated. To develop this figure, we first calculate the probability of dying $P^{(\text {dead})}_{\gamma }$ during the first *γ*>0 time units in the undiagnosed state:

6 $$ \begin{aligned} {}P^{(\text{dead})}_{\gamma} (t, a) &= \int_{0}^{\gamma} \mu_{1} (t + s, a + s)\\ &\exp \left(- \int_{0}^{s} (\lambda_{1} + \mu_{1})(t + \tau, a + \tau) \mathrm{d}\tau \right) \, \mathrm{d}s. \end{aligned}  $$

The probability $P^{(\text {dead})}_{\gamma }$ is combined with the incidence rate *λ*_0_:

7 $$ \Phi_{\gamma}(t,a) = \lambda_{0}(t,a) \, P^{(\text{dead})}_{\gamma} (t,a).  $$

Then, *Φ*_*γ*_(*t*,*a*) is the number of persons aged *a* at time *t*, who become incident undiagnosed cases at *t* and die within *γ* time units without diagnosis. These originally healthy persons never had the chance of obtaining a treatment.

#### Composite indices

In the field of infectious disease epidemiology, the *case detection rate* (CDR) is defined as the notification rate of incident cases over the total incidence rate. Roughly speaking, it is the proportion of *detected incident* cases from the *total incident* cases [8]. In our multi-state model, the total incidence is *λ*_0_ and the CDR can be calculated as:

8 $$ \text{CDR} = \text{DR}_{0} \times \, \omega_{2}.  $$

A proof for this relation can be found in the Appendix. As the CDR includes the transition rate ratio DR_0_ and the prevalence-based index *ω*_2_, we call CDR a *composite index* for assessing case-finding.

Table 1 sums up the different indices for assessing case-finding.

**Table 1:** Summary of all indices for assessing case-finding

Index	Formula	Remark
*ω*_1_	$\tfrac {p_{2}}{p_{1} + p_{2}}$	Percentage of diagnosed over total cases
*ω*_2_	$\tfrac {p_{1}}{p_{0} + p_{1}}$	Inverse of the average number of persons without a diagnosis whom a physician must see in order to meet one undiagnosed case
DR_*γ*_(*t*,*a*)	$\tfrac {\lambda _{1}(t + \gamma, a + \gamma)}{\lambda _{0}(t, a)}$	Rate ratio of diagnosing a person exactly *γ* years after contracting the disease
CDR	DR_0_ *ω*_2_	Case detection ratio
*Φ*_*γ*_(*t*,*a*)	*λ*_0_(*t*,*a*) *P* *γ*(dead)(*t*,*a*)	Number of healthy persons aged *a* at *t* who die with at most *γ* years of undiagnosed disease

### Simulation

Carstensen and co-workers have described an increase of the incidence of diagnosed diabetes in Denmark during the second half of the 1990ies [9]. The data this estimate was based upon stem from a nationwide diabetes register with a catchment population of more than 5 million people. The data about diagnosed diabetes from Denmark are very comprehensive and very detailed. Unfortunately, there are no data of similar high quality and details about undiagnosed diabetes for Denmark during that time. So, we use the rates reported in [9] and additional assumptions to include the state of undiagnosed diabetes in our simulation. The scenarios are hypothetical, because the Danish register does not report data about undiagnosed diabetes. The aim of this work is not to state that the situation of undiagnosed diabetes in Denmark *has been* as we describe, we are only interested in simulating somewhat realistic situations that *could have* been. Our aim is the exploration and comparison of how the indices of case-finding perform in these scenarios. We confine ourselves to the data from the male Danish population.

Together with an initial condition, the simulation uses the rates *λ*_*ℓ*_, *ℓ*=0,1, and *μ*_*k*_, *k*=0,1,2 as described below and integrates Eqs. (1) and (2) to obtain the functions *p*_*k*_, *k*=1,2. We will numerically integrate the system (1)–(2) using the Method of Characteristics [10] and Runge-Kutta numerical integration [11]. After numerically integrating system (1)–(2), we apply the epidemiological indices that have been described above.

#### Initial condition

The age-specific prevalence of diagnosed diabetes in the male Danish population in the year 1995 serves as the initial condition for *p*_2_. Additionally, we assume that the prevalence of undiagnosed diabetes (*p*_1_) is half of *p*_2_.

#### Mortality

The mortality rate *μ*_0_ in our simulation is chosen to be the mortality of the non-diabetic population in Denmark. Following [9], a yearly decrease of 2.5% for all ages is assumed. Because there is evidence that the all-cause mortality of men with undiagnosed diabetes is elevated by 25% as compared to normoglycemic men [12], we assume *μ*_1_=1.25 *μ*_0_. The mortality of men with diagnosed diabetes (*μ*_2_) is taken from the Danish register.

#### Transition rate *λ*_0_

**Fig. 2:**
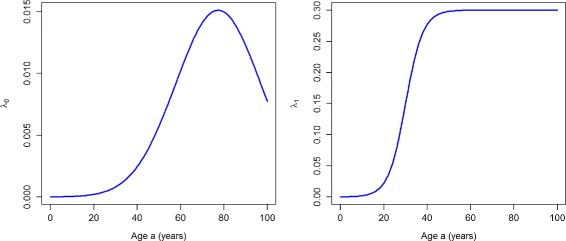
Incidence rates. Age-specific transition rates *λ*_0_ (*left*) and *λ*_1_ (*right*) in year *t*=1995

Detailed information about the incidence of diagnosed diabetes is reported by Carstensen et al. To include a state of undiagnosed diabetes, the transition rate *λ*_0_ from the *Normal* to the *Undiagnosed* state is assumed to be twice as high as the incidence of diagnosed diabetes in [9] (left part of Fig. 2). Although the exact ratio between the incidence of undiagnosed and diagnosed diabetes has never been surveyed, the last assumption is based on the observation that in Denmark there is a considerable proportion of undiagnosed diabetes [13, 14]. Thus, the amount of overall diabetes is higher than diagnosed diabetes alone. The increase of 5.3% per year has again been taken from the Danish register data.

#### Transition rate *λ*_1_

The rate *λ*_1_ is chosen as a sigmoid function (right part of Fig. 2). It mimics an awareness for diabetes, which increases from age 15 until some saturation level at about 40 years of age. After an age of 40 years, the level remains constant. From year 1995 to year 2000, we assume that there is no annual trend in *λ*_1_. During 2000–2005, for *λ*_1_ we assume an annual change of −5*%* and +15*%* in the PU^+^ and the PU^−^ scenarios, respectively. These choices are merely assumptions because we do not have any empirical data about *λ*_1_.

Based on the compartment model shown in Fig. 1 and the data from the Danish register, the indices summarized in Table 1 are applied to the two scenarios PU^+^ and PU^−^. Then, the results are compared. All calculations for this work have been performed with the statistical software R (The R Foundation for Statistical Computing). The R source files for running the simulation and applying the indices for assessing the case-finding are provided as an Additional file 1 to this manuscript.

## Results

### Scenario of increasing prevalence of undiagnosed disease

**Fig. 3:**
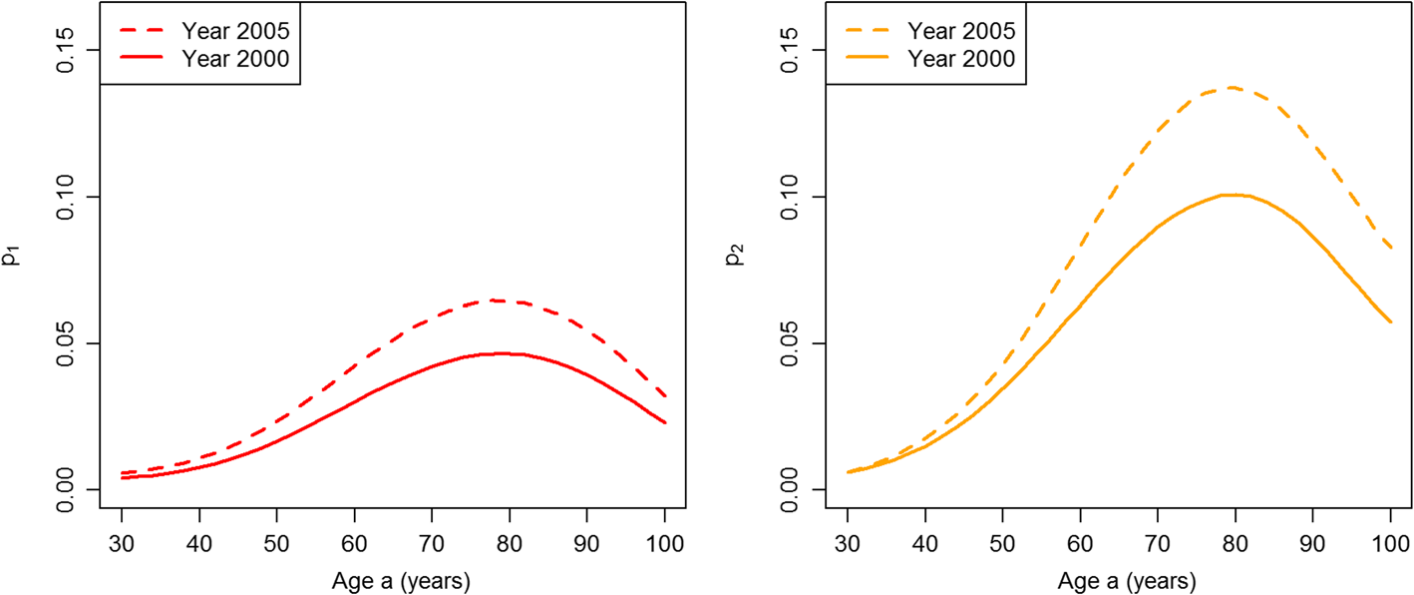
Prevalences in the worsened case-finding scenario. Age-specific prevalences *p*_1_ (*left*) and *p*_2_ (*right*) in the years *t*=2000 (*solid lines*) and *t*=2005 (*dashed lines*) in the scenario PU^+^

We will start with the PU^+^ scenario. Figure 3 shows the age-specific prevalence of the undiagnosed (*p*_1_, left part of Fig. 3) and the diagnosed disease (*p*_2_, right part) in the years *t*_1_=2000 (solid lines) and *t*_2_=2005 (dashed lines). For all ages, there is an increase of the prevalence of undiagnosed diabetes from 2000 to 2005. Thus, in the PU^+^ scenario there is an accumulation of undetected cases in the undiagnosed state. Similarly, for virtually all ages there is an increase of the diagnosed prevalence during the period 2000–2005. If we compare the age-specific prevalences for 2000 and 2005 in the right part of Fig. 3 with the corresponding prevalence data published in [9], we see a good agreement. Hence, the rates chosen for the PU^+^ scenario are consistent with the observed register data from Denmark.

**Fig. 4:**
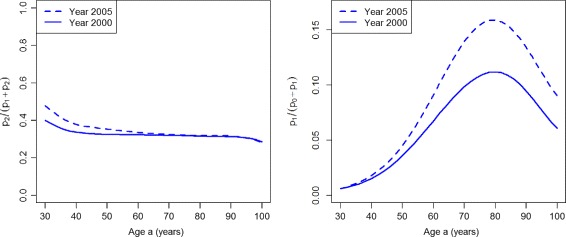
Prevalence-based indices in the worsened case-finding scenario. Age-specific indices of case-finding *ω*_1_ (*left*) and *ω*_2_ (*right*) in the years *t*=2000 (*solid lines*) and *t*=2005 (*dashed lines*) in the scenario PU^+^

If we calculate the prevalence-based indices of case-finding *ω*_1_ and *ω*_2_, we obtain the graphs shown in Fig. 4. The left part of Fig. 4 shows that *ω*_1_ indicates an improvement of case-finding between the years 2000 (solid line) and 2005 (dashed line) for the age range 30 to about 60. For higher ages the two lines converge, which indicates no difference in the performance of case-finding.

The index *ω*_2_ (right part of Fig. 4) finds that case-finding worsens during the five years from 2000 to 2005 for all ages older than 40 years. For lower ages, the index does not show a difference between the years 2000 and 2005.

**Fig. 5:**
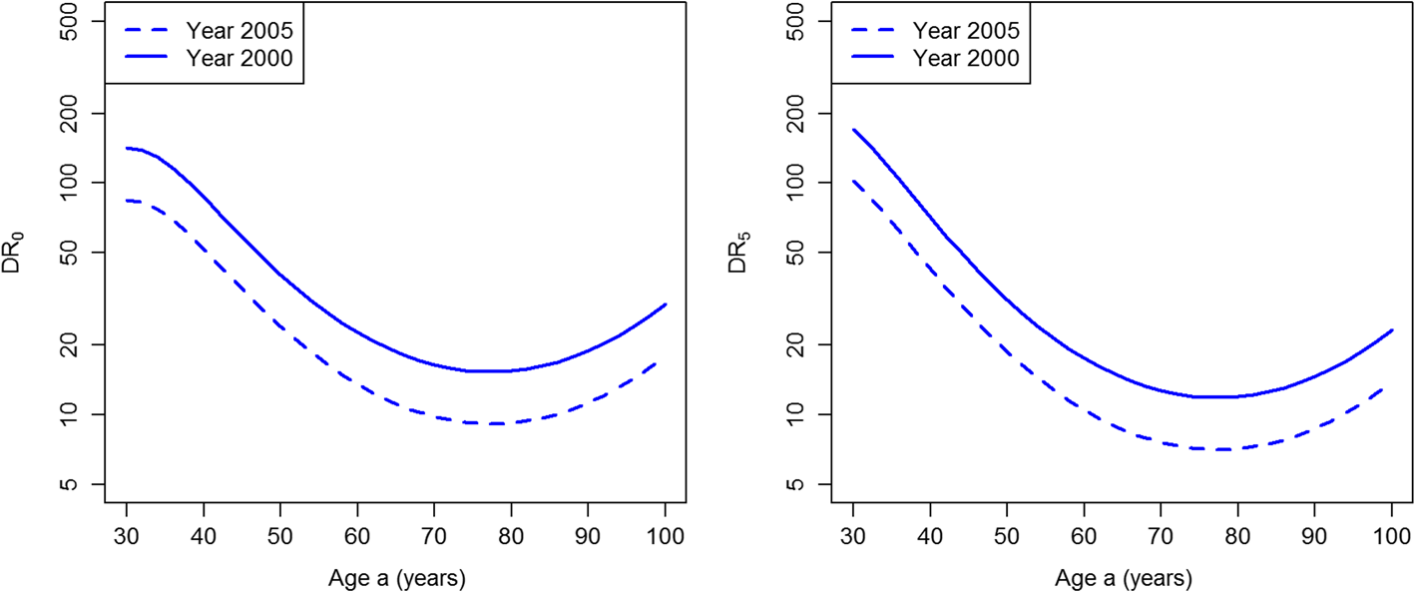
Detection ratios in the worsened case-finding scenario. Age-specific detection ratios DR_0_ (*left*) and DR_5_ (*right*) in the years *t*=2000 (*solid lines*) and *t*=2005 (*dashed lines*) in the scenario PU^+^

Figure 5 shows the indices based on the incidence rates. For the whole age range, both indices display a decreasing performance of case-finding during the period 2000 and 2005.

**Fig. 6:**
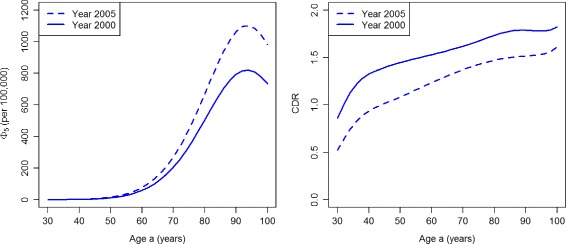
Number of deaths from the undiagnosed state and case detection rate in the worsened case-finding scenario. Age-specific number *Φ*_5_ (per 100,000 healthy persons, *left*) and the case detection rate CDR (*right*) in the years *t*=2000 (*solid lines*) and *t*=2005 (*dashed lines*) in the scenario PU^+^

Similarly, the indices *Φ*_5_ and CDR (Fig. 6) show a decreasing performance of case-finding for all ages during 2000–2005. Note the large increase in the numbers of persons dying without ever obtaining a treatment. For example, consider 100,000 healthy persons aged 90 in year 2000. Of these, 1587 develop diabetes during that year. During the next five years, 795 (50%) of these 1587 die without ever obtaining a diagnosis. In 2005, 2055 persons develop diabetes and 1064 (52%) of these die without a diagnosis during the next five years.

### Scenario of decreasing prevalence of undiagnosed disease

**Fig. 7:**
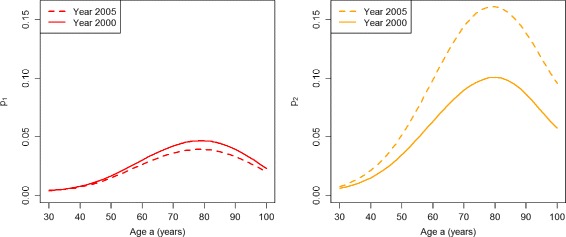
Prevalences in the improved case-finding scenario. Age-specific prevalences *p*_1_ (*left*) and *p*_2_ (*right*) in the years *t*=2000 (*solid lines*) and *t*=2005 (*dashed lines*) in the scenario PU^−^

In the PU^−^ scenario, we obtain the age-specific prevalences *p*_1_ and *p*_2_ in the years *t*_1_=2000 (solid lines) and *t*_2_=2005 (dashed lines) as shown in Fig. 7.

For all ages above about 45, there is a decrease of the prevalence of undiagnosed diabetes during the period from 2000 to 2005. The age-specific prevalences of undiagnosed and diagnosed diabetes in year 2000 agree with the corresponding curves in the PU^+^ scenario (see Fig. 3). For virtually all ages, there is a decrease of the prevalence of undiagnosed diabetes during the period 2000–05. During the same period the prevalence of the diagnosed disease increases for all ages.

**Fig. 8:**
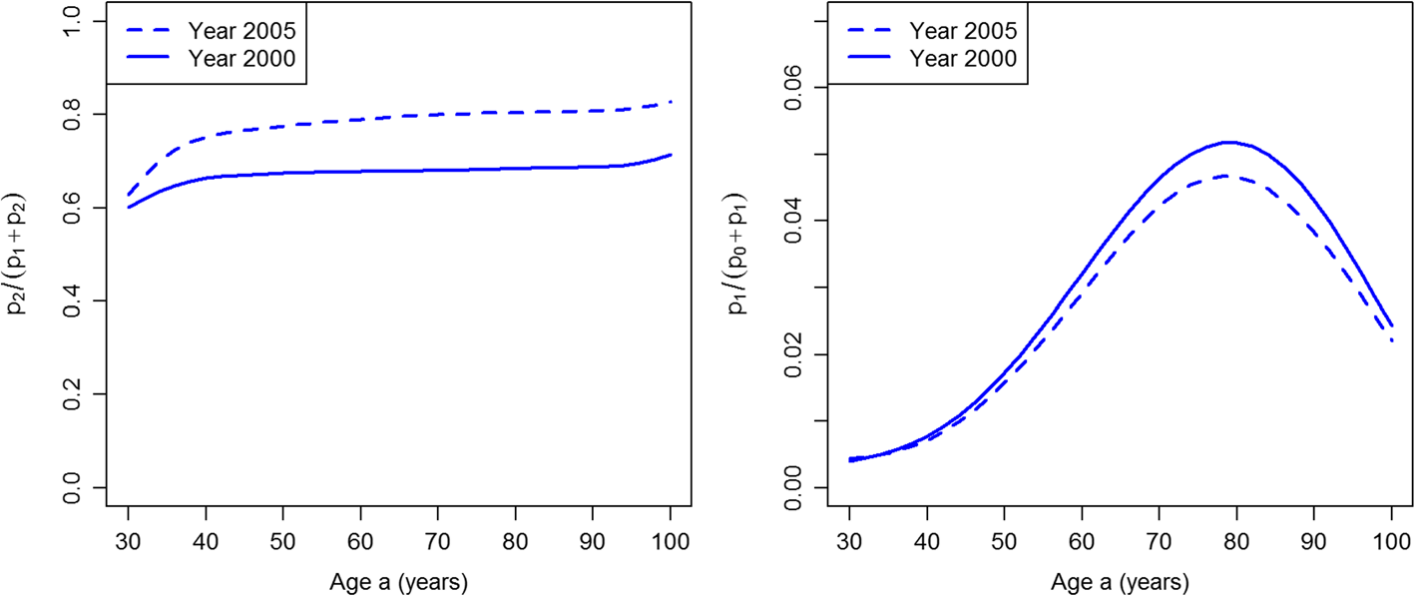
Prevalence-based indices in the improved case-finding scenario. Age-specific indices of case-finding *ω*_1_ (*left*) and *ω*_2_ (*right*) in the years *t*=2000 (*solid lines*) and *t*=2005 (*dashed lines*) in the scenario PU^−^

The prevalence-based indices *ω*_1_ and *ω*_2_ are shown in Fig. 8. Both indices *ω*_1_ and *ω*_2_ indicate an improvement of the quality case-finding.

**Fig. 9:**
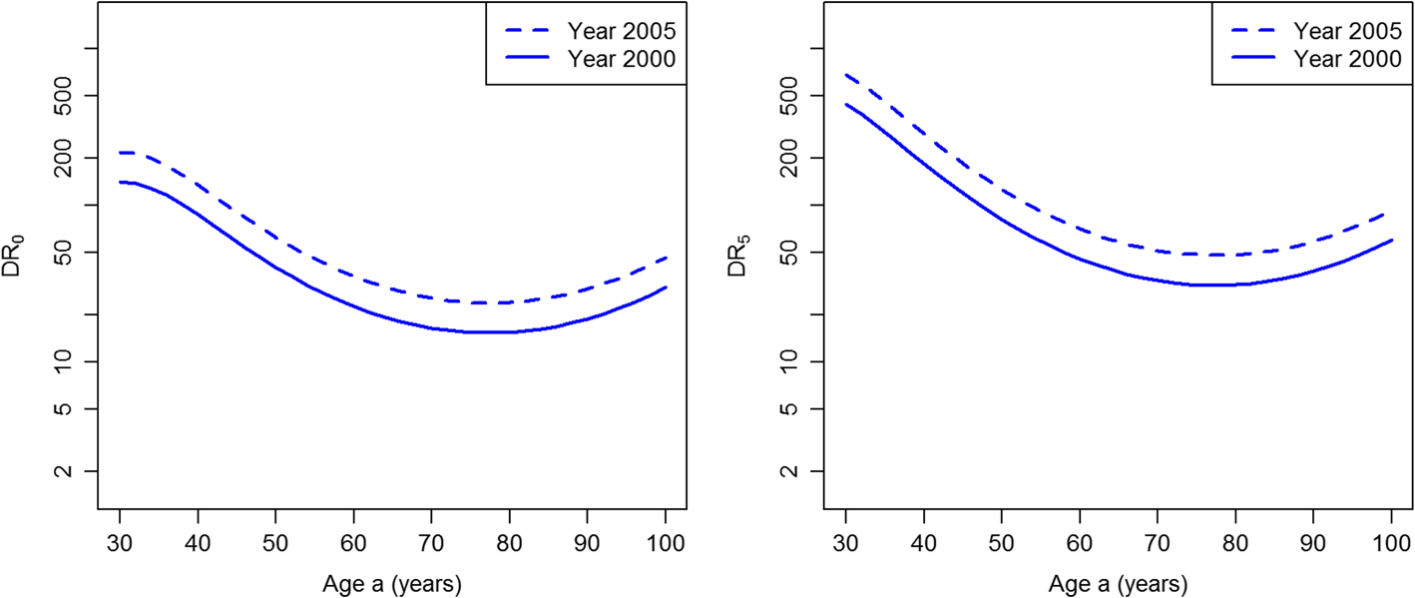
Detection ratios in the improved case-finding scenario. Age-specific detection ratios DR_0_ (*left*) and DR_5_ (*right*) in the years *t*=2000 (*solid lines*) and *t*=2005 (*dashed lines*) in the scenario PU^−^

**Fig. 10:**
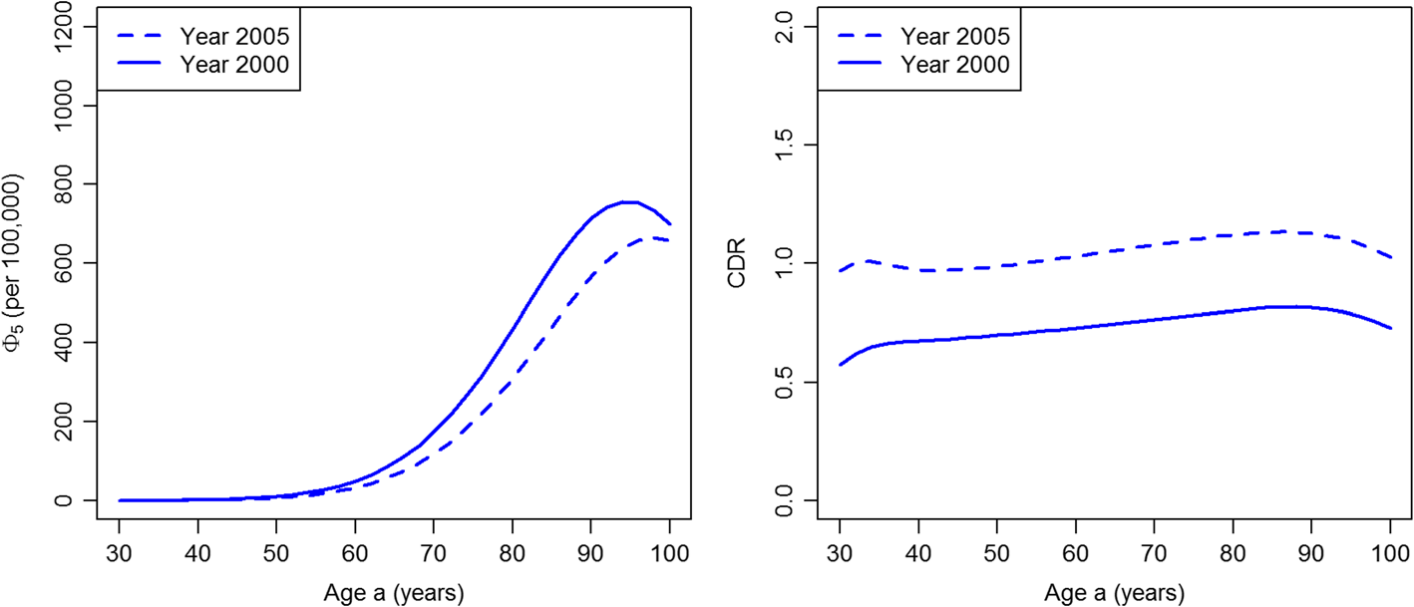
Number of deaths from the undiagnosed state and case detection rate in the improved case-finding scenario. Age-specific number *Φ*_5_ (per 100,000 healthy persons, *left*) and the case detection rate CDR (*right*) in the years *t*=2000 (*solid lines*) and *t*=2005 (*dashed lines*) in scenario PU^−^

The ratios DR_0_ and DR_5_ are presented in Fig. 9. For the whole age range, both indices DR_0_ and DR_5_ consistently display an increasing performance of case-finding during the considered period. The same holds true for the indices CDR and *Φ*_5_ (Fig. 10)

For comparison with the PU^+^ scenario, consider 100,000 healthy persons aged 90 in year 2000. Again, 1587 develop diabetes during that year. During the next five years, 713 (45%) of these 1587 die without obtaining a diagnosis. In 2005, from those 2055 persons who become diabetic in that year 566 (28%) die without a diagnosis during the next five years. This is a substantial reduction, which indicates an improvement in case-finding.

Table 2 sums up the findings of assessing the performance of case-finding in the different simulations scenarios. The index *ω*_1_ at least partly contradicts the findings of the other indices in scenario PU^+^, whereas the figures *p*_1_, *ω*_2_, DR_0_, DR_5_ and *Φ*_5_ assess both scenarios consistently. For some ages, the indices *p*_1_ and *Φ*_5_ are indecisive.

**Table 2:** Summary of the different indices of assessing case-finding

	Prevalence of undiagnosed disease	
Index	Increasing (PU^+^)	Decreasing (PU^−^)
*ω*_1_	(+)	+
*ω*_2_	–	+
DR_0_	–	+
DR_5_	–	+
CDR	–	+
*Φ*_5_	(–)	(+)

A plus (+) or a minus (–) sign denotes whether the index indicates an improvement or a deterioration of case-finding over time. If a plus or minus sign is put in parentheses, the index is indecisive for some ages

## Discussion

Based on a recently developed multi-state model, we simulated two hypothetical scenarios about trends in undiagnosed type 2 diabetes. In one scenario the incidence rate of diagnoses decreased over time reflecting a worsened case-finding. In the other scenario, the performance of case-finding improved. Several indices for assessing case-finding have been applied to the two scenarios.

We found that the measure *ω*_1_ leads to an inconsistent assessment of the case-finding performance in our scenarios. In the scenario of increasing prevalence of undiagnosed disease (PU^+^), *ω*_1_ indicates an improvement whereas all other indices indicate a worsening of the quality of case-finding.

The index *ω*_2_ assesses both scenarios according to the incidence-based and composite indices. Thus, *ω*_2_ is a sensitive measure for the improvement and degradation of case-finding in our simulation. There is a theoretical reason for the appropriateness of *ω*_2_. As we have seen in Eq. (5), the expression $\frac {p_{0}}{p_{1}} + \frac {\mu -\mu _{1}}{\lambda _{0}}$ determines if *p*_1_ is increasing or decreasing. This expression can be reformulated in terms of *ω*_2_:

$$\frac{p_{0}}{p_{1}} + \frac{\mu-\mu_{1}}{\lambda_{0}} = \frac{1}{\omega_{2}} - 1 + \frac{\mu-\mu_{1}}{\lambda_{0}}. $$

Thus, we can see that *ω*_2_ plays a direct role in determining if the prevalence of the undiagnosed disease is increasing or decreasing.

Similarly, the detection ratios DR_*γ*_ assess the different simulation scenarios for *γ*=0 and *γ*=5 consistently with the other indices (except for *ω*_1_). The figure *Φ*_5_ is an important measure, which refers to a cohort of healthy persons who contract the disease but never get the chance of being treated.

To our knowledge, the disease model in Fig. 1 has only been reported in [7]. In contrast to existing state models with a compartment preceding the diagnosis, typically called *preclinical* state [15, 16], our model includes the possibility of dying from the undiagnosed (preclinical) state. In diabetes, there is a considerably increased mortality from this state [12].

In the literature, there is another index to assess case-finding, the mean sojourn time (MST) in the preclinical phase (see [17] for a review). Usually, a low MST is considered advantageous. However, the MST may be low if the mortality from the undiagnosed state is high. Thus, the MST is not an appropriate figure for evaluating case-finding if there is considerable mortality from the undiagnosed (or preclinical) state. Hence, the MST has not been considered in this work.

Apart from the systematic comparison of the different indices, we introduced two new indices *ω*_2_ and *Φ*_*γ*_. The later quantifies the number of healthy persons who become incident undiagnosed cases and die before ever obtaining a diagnosis. In populations with a high incidence (i.e., large *λ*_0_) and a poor case-finding (low *λ*_1_), the number *Φ*_*γ*_ will be high. This can be observed in the two simulation scenarios. While in the PU^+^ scenario *Φ*_5_ increases from 2000 to 2005, there is a large decrease of *Φ*_5_ in the PU^−^ scenario. In such cases, it would be interesting to compare this index for different strata of people in the considered population, e.g., low vs. high age, or low versus high socio-economic status.

Compared to the other figures, the measure $\omega _{2} = \tfrac {p_{1}}{p_{0}+p_{1}}$ has the advantage that it requires prevalence data only. Prevalences can be obtained from cross-sectional studies. Those measures that include the incidence rates either require costly follow-up data or the application of specialized estimation techniques [7].

The question arises, how the different indices may be surveyed. The prevalence-based metrics *ω*_1_ and *ω*_2_ can be estimated from cross-sectional surveys that comprise estimation of diagnosed *and undiagnosed* disease. A variety of cross-sectional studies ask participants about prior diagnoses, for instance, in case of diabetes [18–20] or hypertension [21].

The indices based on the transition rates can be estimated by studies with follow-up information such as cohort studies. A practical demonstration using data from the *Health and Retirement Study* has been described in a recent manuscript [7], which also illustrates how to deal with statistical uncertainty.

Although the data and examples of this manuscript are given primarily for diabetes, the presented concepts can be applied in other chronic diseases like those mentioned in the introduction. For example, the estimation of *ω*_1_ and *ω*_2_ in a nationally representative study [21] about hypertension is straight forward.

## Conclusion

This article compiles and compares several indices for case-finding in the field of chronic diseases. To our knowledge, this is the first systematic comparison of indirect surveillance-based metrics for the monitoring of case finding. We found that in assessing case-finding, incidence-based metrics should be preferred, because they gain a deeper insight into the dynamics of the compartment system than prevalence-based indices. Eqs. (1)–(2) show that the prevalences *p*_*k*_, *k*=1,2, depend on all transition rates in the multi-state model, on the incidence rates *λ*_*ℓ*_, *ℓ*=0,1, and also on the mortality rates *μ*_*k*_(*t*,*a*), *k*=0,1,2. In this sense, the prevalences are the result of a complex interplay of the transition rates, both incidence and mortality rates.

Thus, prevalence-based indices of case-finding should be interpreted with caution, because they may lead to inconsistent findings (*ω*_1_). If prevalence-based metrics have to be used, the new index *ω*_2_ is preferable over *ω*_1_, because *ω*_2_ has shown to be the better index to distinguish the simulated scenarios of increasing or decreasing prevalence of undiagnosed disease.

Indices based on transition rates provide more insights into the system. The detection ratio (DR) provides a necessary and sufficient condition whether the prevalence of undiagnosed disease is increasing or decreasing. Moreover, only the true incidence rate *λ*_0_ indicates if the distributions of the underlying risk factors of the considered chronic disease changes. Thus, if the incidence of a chronic disease is used for surveillance, it is important to always consider the *Undiagnosed* state. If this state is ignored, and instead the combined state of *Normal* and *Undiagnosed* is considered, the rate *λ* ^′^ surveyed (see Appendix). Changes in the rate *λ*_1_ reflect societal processes like medical progress and changes in patients’ or physicians’ awareness.

In summary, these simulation analyses identify potential pitfalls of commonly-used indirect measures of case detection. These findings suggest that the *ω*_2_ term (proportion of all persons without a diagnosis who have the disease) is a preferable index to the *ω*_1_ term (proportion of all cases who have been diagnosed). However, these findings also underscore the need to use more direct estimates of incidence itself and to incorporate more direct estimates of detection into ongoing chronic disease surveillance systems.

## Appendix

The CDR is the proportion of incident cases being diagnosed [8], i.e., the notification rate over the total incidence rate. Let *λ* ^′^ be the notification rate, which is the number of detected cases transiting from the search space to the *Diagnosed* state per unit time. Thus, the denominator of *λ* ^′^ is the number of persons in the combined state of *Normal* and *Undiagnosed*. In Fig. 1, the rate *λ*_1_ refers to transitions from the *Undiagnosed* state to the *Diagnosed* state. Here, the denominator is the number of persons in the *Undiagnosed* state. Hence, it holds $\lambda ' = \tfrac {p_{1}}{p_{0} + p_{1}} \, \lambda _{1}.$ As *λ*_0_ is the overall incidence, it holds

$$\text{CDR} = \frac{p_{1} \, \lambda_{1}}{(p_{0} + p_{1}) \lambda_{0}} = \omega_{2} \, \text{DR}_{0}. $$

## Additional file

Additional file 1 Scripts for the statistical software R. The zip-file contains the analysis for the simulation. For detailed instructions unzip the file and refer to the readme.txt file. (ZIP 4 kb)

## Abbreviations

CDR: Case detection rate
DR: Detection rate ratio
PU^−^: Improving case-finding
PU^+^: Worsening case-finding
